# Supplementary material for: Splice-Junction-Based Mapping of Alternative Isoforms in the Human Proteome
Source: Cell Rep. Author manuscript; Available in PMC 2020 Jan 15. (PMC6961840; doi:10.1016/j.celrep.2019.11.026)
Supplement: 2 [file NIHMS1546469-supplement-2.zip › DF1/PXD006675/AtrialSeptum/AtrialSeptum_24_RYR2_VLDIANVLFHLEQVEHPQR.pdf]

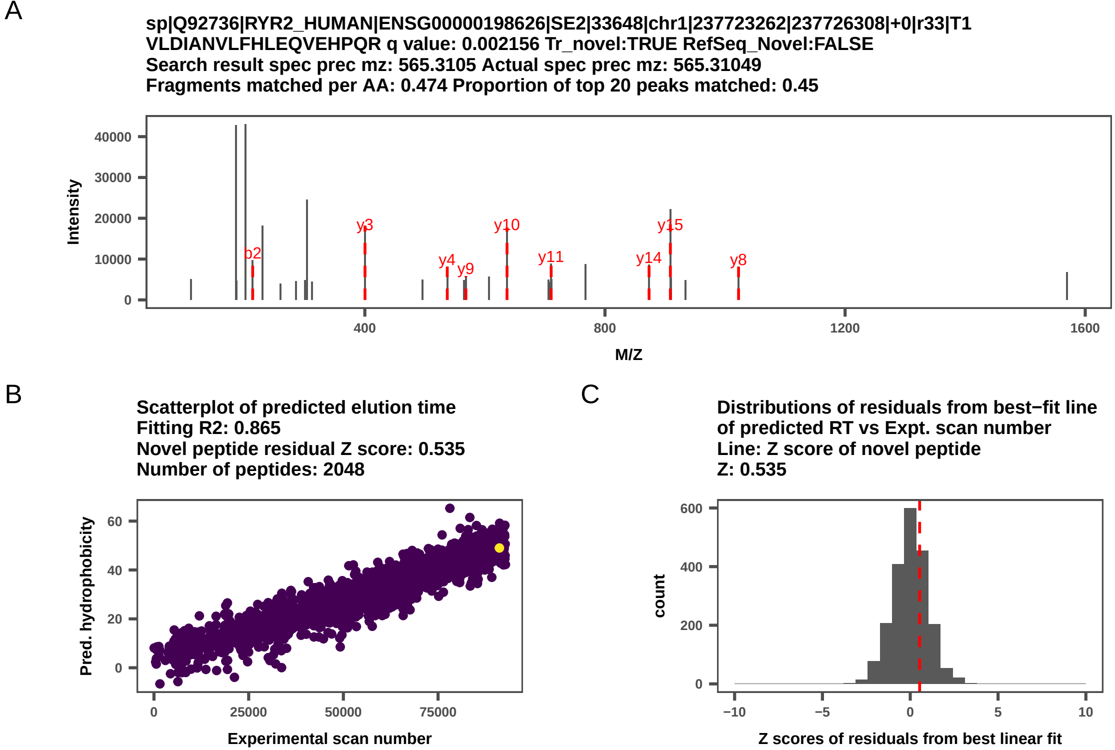
A
sp|Q92736|RYR2_HUMAN|ENSG00000198626|SE2|33648|chr1|237723262|237726308|+0|r33|T1
VLDIANVLFHLEQVEHPQR q value: 0.002156 Tr_novel:TRUE RefSeq_Novel:FALSE
Search result spec prec mz: 565.3105 Actual spec prec mz: 565.31049
Fragments matched per AA: 0.474 Proportion of top 20 peaks matched: 0.45
Intensity
M/Z
b2
y3
y4
y9
y10
y11
y14
y15
y8
B
Scatterplot of predicted elution time
Fitting R2: 0.865
Novel peptide residual Z score: 0.535
Number of peptides: 2048
Pred. hydrophobicity
Experimental scan number
C
Distributions of residuals from best−fit line
of predicted RT vs Expt. scan number
Line: Z score of novel peptide
Z: 0.535
count
Z scores of residuals from best linear fit
